# 5-(3,4-Dimethyl­benzyl­idene)-2,2-dimethyl-1,3-dioxane-4,6-dione

**DOI:** 10.1107/S1600536811016497

**Published:** 2011-05-07

**Authors:** Wu-Lan Zeng

**Affiliations:** a MicroScale Science Institute, Department of Chemistry and Chemical Engineering, Weifang University, Weifang 261061, People’s Republic of China

## Abstract

The title compound, C_15_H_16_O_4_, was prepared by the reaction of 2,2-dimethyl-1,3-dioxane-4,6-dione and 3,4-dimethyl­benzaldehyde in ethanol. The 1,3-dioxane ring exhibits an envelope conformation. In the crystal, mol­ecules are linked by weak inter­molecular C—H⋯O hydrogen bonds, forming chains parallel to the *b* axis.

## Related literature

For related structures, see: Zeng (2010 ▶, 2011 ▶).
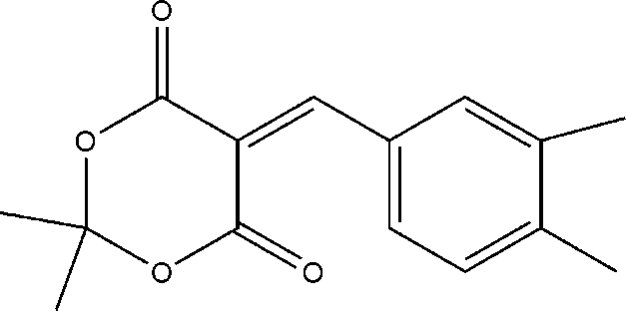


## Experimental

### 

#### Crystal data

C_15_H_16_O_4_
*M* *_r_* = 260.28
Monoclinic, 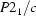

*a* = 16.8249 (15) Å
*b* = 7.1390 (6) Å
*c* = 11.7101 (11) Å
β = 108.612 (1)°
*V* = 1333.0 (2) Å^3^
*Z* = 4
Mo *K*α radiation
μ = 0.09 mm^−1^
*T* = 298 K
0.45 × 0.32 × 0.30 mm

#### Data collection

Bruker SMART CCD area-detector diffractometer
Absorption correction: multi-scan (*SADABS*; Bruker, 1997 ▶) *T*_min_ = 0.959, *T*_max_ = 0.972
6611 measured reflections
2341 independent reflections
1330 reflections with *I* > 2σ(*I*)
*R*_int_ = 0.040

#### Refinement

*R*[*F* ^2^ > 2σ(*F* ^2^)] = 0.048
*wR*(*F* ^2^) = 0.201
*S* = 1.09
2341 reflections
176 parameters
H-atom parameters constrained
Δρ_max_ = 0.15 e Å^−3^
Δρ_min_ = −0.15 e Å^−3^

### 

Data collection: *SMART* (Bruker, 1997 ▶); cell refinement: *SAINT* (Bruker, 1997 ▶); data reduction: *SAINT*; program(s) used to solve structure: *SHELXS97* (Sheldrick, 2008 ▶); program(s) used to refine structure: *SHELXL97* (Sheldrick, 2008 ▶); molecular graphics: *SHELXTL* (Sheldrick, 2008 ▶); software used to prepare material for publication: *SHELXTL*.

## Supplementary Material

Crystal structure: contains datablocks global, I. DOI: 10.1107/S1600536811016497/rz2588sup1.cif

Structure factors: contains datablocks I. DOI: 10.1107/S1600536811016497/rz2588Isup2.hkl

Supplementary material file. DOI: 10.1107/S1600536811016497/rz2588Isup3.cml

Additional supplementary materials:  crystallographic information; 3D view; checkCIF report

## Figures and Tables

**Table 1:** Hydrogen-bond geometry (Å, °)

*D*—H⋯*A*	*D*—H	H⋯*A*	*D*⋯*A*	*D*—H⋯*A*
C6—H6*C*⋯O4^i^	0.96	2.58	3.447 (4)	151

Symmetry code: (i) 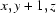.

**Fig. 1.**
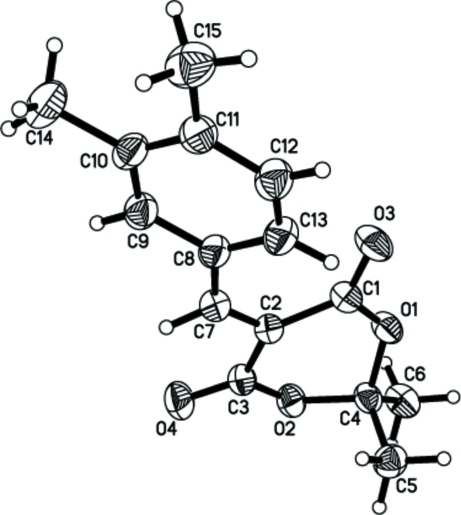
The molecular structure of the title compound, with displacement ellipsoids drawn at the 30% probability level.

## supplementary crystallographic information

### Comment

In previous papers, the crystal structure of 5-(4-hydroxybenzylidene)-2,2-dimethyl-1,3-dioxane-4,6-dione (Zeng, 2010) and 2,2-dimethyl-5-[(5-methylfuran-2-yl)methylidene]-1,3-dioxane-4,6-dione (Zeng, 2011) have been reported. As part of this ongoing search for new Meldrum's acid compounds, the title compound  has been synthesized and its structure is reported here.

In the title compound (Fig. 1), bond lengths and angles fall in the usual ranges. The 1,3-dioxane ring exhibits an envelope conformation with the dimethyl-substituted carbon C4 atom forming the flap. In the crystal structure, the molecules interact through a weak intermolecular C—H···O hydrogen bond (Table 1) to form chains parallel to the *b* axis.

### Experimental

The mixture of malonic acid (6.24 g, 0.06 mol) and acetic anhydride (9 ml) in concentrated sulfuric acid (0.25 ml) was stirred with water at 303K. After dissolving, propan-2-one (3.48 g, 0.06 mol) was added dropwise into the solution and the reaction was allowed to proceed for 2 h. The mixture was then cooled and filtered, and an ethanol solution of 3,4-dimethylbenzaldehyde (8.04 g, 0.06 mol) was added. The solution was then filtered and concentrated. Single crystals were obtained by slow evaporation of a petroleum ether/ethylacetate (4:1 *v*/*v*) solution at room temperature over a period of several days.

### Refinement

The H atoms were placed in calculated positions (C—H = 0.93–0.96 Å), and refined as riding with *U*_iso_(H) = 1.2 *U*_eq_(C) or 1.5 U_eq_(C) for methyl H atoms.

### Crystal data

**Table:**

C_15_H_16_O_4_	*F*(000) = 552
*M**_r_* = 260.28	*D*_x_ = 1.297 Mg m^−^^3^
Monoclinic, *P*2_1_/*c*	Mo *K*α radiation, λ = 0.71073 Å
Hall symbol: -P 2ybc	Cell parameters from 1211 reflections
*a* = 16.8249 (15) Å	θ = 2.6–21.6°
*b* = 7.1390 (6) Å	µ = 0.09 mm^−^^1^
*c* = 11.7101 (11) Å	*T* = 298 K
β = 108.612 (1)°	Block, yellow
*V* = 1333.0 (2) Å^3^	0.45 × 0.32 × 0.30 mm
*Z* = 4	

### Data collection

**Table:**

Bruker SMART CCD area-detector diffractometer	2341 independent reflections
Radiation source: fine-focus sealed tube	1330 reflections with *I* > 2σ(*I*)
graphite	*R*_int_ = 0.040
φ and ω scans	θ_max_ = 25.0°, θ_min_ = 2.6°
Absorption correction: multi-scan (*SADABS*; Bruker, 1997)	*h* = −19→20
*T*_min_ = 0.959, *T*_max_ = 0.972	*k* = −8→8
6611 measured reflections	*l* = −13→11

### Refinement

**Table:**

Refinement on *F*^2^	Primary atom site location: structure-invariant direct methods
Least-squares matrix: full	Secondary atom site location: difference Fourier map
*R*[*F*^2^ > 2σ(*F*^2^)] = 0.048	Hydrogen site location: inferred from neighbouring sites
*wR*(*F*^2^) = 0.201	H-atom parameters constrained
*S* = 1.09	*w* = 1/[σ^2^(*F*_o_^2^) + (0.0016*P*)^2^] where *P* = (*F*_o_^2^ + 2*F*_c_^2^)/3
2341 reflections	(Δ/σ)_max_ < 0.001
176 parameters	Δρ_max_ = 0.15 e Å^−^^3^
0 restraints	Δρ_min_ = −0.15 e Å^−^^3^

### Special details

**Table:**

Geometry. All e.s.d.'s (except the e.s.d. in the dihedral angle between two l.s. planes) are estimated using the full covariance matrix. The cell e.s.d.'s are taken into account individually in the estimation of e.s.d.'s in distances, angles and torsion angles; correlations between e.s.d.'s in cell parameters are only used when they are defined by crystal symmetry. An approximate (isotropic) treatment of cell e.s.d.'s is used for estimating e.s.d.'s involving l.s. planes.
Refinement. Refinement of *F*^2^ against ALL reflections. The weighted *R*-factor *wR* and goodness of fit *S* are based on *F*^2^, conventional *R*-factors *R* are based on *F*, with *F* set to zero for negative *F*^2^. The threshold expression of *F*^2^ > σ(*F*^2^) is used only for calculating *R*-factors(gt) *etc*. and is not relevant to the choice of reflections for refinement. *R*-factors based on *F*^2^ are statistically about twice as large as those based on *F*, and *R*- factors based on ALL data will be even larger.

### Fractional atomic coordinates and isotropic or equivalent isotropic displacement parameters (Å^2^)

**Table:**

	*x*	*y*	*z*	*U*_iso_*/*U*_eq_	
O1	0.30755 (12)	0.6940 (3)	0.53926 (17)	0.0576 (6)	
O2	0.39334 (12)	0.4548 (3)	0.50624 (17)	0.0579 (6)	
O3	0.21388 (14)	0.6469 (3)	0.62839 (19)	0.0710 (7)	
O4	0.39031 (14)	0.1691 (3)	0.5720 (2)	0.0791 (8)	
C1	0.27252 (18)	0.5845 (4)	0.6047 (3)	0.0523 (8)	
C2	0.30722 (17)	0.3945 (4)	0.6311 (3)	0.0518 (8)	
C3	0.36633 (19)	0.3278 (4)	0.5704 (3)	0.0554 (8)	
C4	0.38899 (17)	0.6503 (4)	0.5324 (3)	0.0508 (8)	
C5	0.45580 (18)	0.6974 (5)	0.6484 (3)	0.0626 (9)	
H5A	0.4516	0.8273	0.6669	0.094*	
H5B	0.5100	0.6735	0.6406	0.094*	
H5C	0.4486	0.6216	0.7121	0.094*	
C6	0.3971 (2)	0.7532 (5)	0.4258 (3)	0.0675 (9)	
H6A	0.3536	0.7135	0.3546	0.101*	
H6B	0.4509	0.7270	0.4172	0.101*	
H6C	0.3921	0.8854	0.4370	0.101*	
C7	0.28095 (18)	0.2646 (4)	0.6950 (3)	0.0603 (9)	
H7	0.3045	0.1474	0.6928	0.072*	
C8	0.22436 (18)	0.2659 (4)	0.7661 (3)	0.0564 (8)	
C9	0.18336 (19)	0.0988 (4)	0.7737 (3)	0.0618 (9)	
H9	0.1947	−0.0067	0.7349	0.074*	
C10	0.12658 (19)	0.0834 (5)	0.8362 (3)	0.0616 (9)	
C11	0.11383 (19)	0.2368 (5)	0.9012 (3)	0.0629 (9)	
C12	0.1564 (2)	0.3997 (5)	0.8989 (3)	0.0676 (9)	
H12	0.1492	0.5014	0.9443	0.081*	
C13	0.20958 (19)	0.4168 (5)	0.8313 (3)	0.0657 (9)	
H13	0.2359	0.5308	0.8292	0.079*	
C14	0.0815 (2)	−0.1001 (5)	0.8349 (3)	0.0894 (12)	
H14A	0.1023	−0.1921	0.7918	0.134*	
H14B	0.0225	−0.0828	0.7960	0.134*	
H14C	0.0913	−0.1419	0.9162	0.134*	
C15	0.0553 (2)	0.2249 (6)	0.9743 (3)	0.0873 (12)	
H15A	0.0004	0.1895	0.9231	0.131*	
H15B	0.0523	0.3446	1.0101	0.131*	
H15C	0.0756	0.1329	1.0367	0.131*	

### Atomic displacement parameters (Å^2^)

**Table:**

	*U*^11^	*U*^22^	*U*^33^	*U*^12^	*U*^13^	*U*^23^
O1	0.0568 (12)	0.0537 (13)	0.0679 (13)	0.0146 (10)	0.0280 (10)	0.0096 (10)
O2	0.0655 (13)	0.0499 (13)	0.0608 (13)	0.0058 (10)	0.0238 (10)	−0.0083 (11)
O3	0.0672 (14)	0.0724 (17)	0.0842 (16)	0.0231 (12)	0.0395 (13)	0.0142 (12)
O4	0.0819 (17)	0.0485 (15)	0.113 (2)	0.0095 (12)	0.0404 (14)	−0.0078 (13)
C1	0.0512 (18)	0.054 (2)	0.0523 (17)	0.0059 (15)	0.0178 (14)	0.0017 (15)
C2	0.0458 (16)	0.0447 (18)	0.0641 (19)	0.0000 (13)	0.0164 (14)	−0.0030 (15)
C3	0.0544 (18)	0.045 (2)	0.066 (2)	0.0024 (15)	0.0183 (15)	−0.0080 (16)
C4	0.0535 (18)	0.0467 (19)	0.0570 (18)	0.0075 (14)	0.0241 (14)	−0.0031 (14)
C5	0.063 (2)	0.065 (2)	0.059 (2)	−0.0037 (16)	0.0175 (16)	−0.0079 (16)
C6	0.071 (2)	0.074 (2)	0.066 (2)	0.0139 (17)	0.0331 (16)	0.0138 (18)
C7	0.0519 (18)	0.0481 (19)	0.076 (2)	0.0015 (15)	0.0136 (16)	0.0016 (16)
C8	0.0520 (18)	0.048 (2)	0.065 (2)	−0.0037 (15)	0.0132 (15)	0.0094 (16)
C9	0.063 (2)	0.053 (2)	0.063 (2)	−0.0030 (15)	0.0103 (16)	0.0072 (15)
C10	0.0559 (19)	0.057 (2)	0.063 (2)	−0.0132 (15)	0.0063 (16)	0.0142 (17)
C11	0.059 (2)	0.061 (2)	0.063 (2)	0.0000 (17)	0.0122 (16)	0.0134 (18)
C12	0.074 (2)	0.060 (2)	0.070 (2)	−0.0043 (17)	0.0241 (18)	0.0107 (17)
C13	0.066 (2)	0.054 (2)	0.077 (2)	−0.0098 (16)	0.0234 (18)	0.0074 (17)
C14	0.093 (3)	0.078 (3)	0.087 (3)	−0.032 (2)	0.015 (2)	0.007 (2)
C15	0.083 (3)	0.099 (3)	0.086 (3)	−0.010 (2)	0.035 (2)	0.014 (2)

### Geometric parameters (Å, °)

**Table:**

O1—C1	1.354 (3)	C7—H7	0.9300
O1—C4	1.432 (3)	C8—C13	1.388 (4)
O2—C3	1.346 (4)	C8—C9	1.395 (4)
O2—C4	1.436 (3)	C9—C10	1.381 (4)
O3—C1	1.193 (3)	C9—H9	0.9300
O4—C3	1.201 (3)	C10—C11	1.389 (4)
C1—C2	1.470 (4)	C10—C14	1.511 (4)
C2—C7	1.351 (4)	C11—C12	1.371 (4)
C2—C3	1.474 (4)	C11—C15	1.500 (4)
C4—C6	1.492 (4)	C12—C13	1.377 (4)
C4—C5	1.499 (4)	C12—H12	0.9300
C5—H5A	0.9600	C13—H13	0.9300
C5—H5B	0.9600	C14—H14A	0.9600
C5—H5C	0.9600	C14—H14B	0.9600
C6—H6A	0.9600	C14—H14C	0.9600
C6—H6B	0.9600	C15—H15A	0.9600
C6—H6C	0.9600	C15—H15B	0.9600
C7—C8	1.452 (4)	C15—H15C	0.9600
			
C1—O1—C4	120.2 (2)	C8—C7—H7	112.6
C3—O2—C4	119.1 (2)	C13—C8—C9	116.8 (3)
O3—C1—O1	117.2 (3)	C13—C8—C7	125.9 (3)
O3—C1—C2	126.7 (3)	C9—C8—C7	117.3 (3)
O1—C1—C2	115.8 (3)	C10—C9—C8	122.7 (3)
C7—C2—C1	124.8 (3)	C10—C9—H9	118.6
C7—C2—C3	115.8 (3)	C8—C9—H9	118.6
C1—C2—C3	118.7 (3)	C9—C10—C11	118.8 (3)
O4—C3—O2	118.2 (3)	C9—C10—C14	119.5 (3)
O4—C3—C2	124.9 (3)	C11—C10—C14	121.6 (3)
O2—C3—C2	116.9 (3)	C12—C11—C10	119.0 (3)
O1—C4—O2	109.8 (2)	C12—C11—C15	120.1 (3)
O1—C4—C6	106.6 (2)	C10—C11—C15	120.9 (3)
O2—C4—C6	106.0 (2)	C11—C12—C13	121.7 (3)
O1—C4—C5	110.7 (2)	C11—C12—H12	119.1
O2—C4—C5	109.7 (2)	C13—C12—H12	119.1
C6—C4—C5	113.9 (3)	C12—C13—C8	120.7 (3)
C4—C5—H5A	109.5	C12—C13—H13	119.6
C4—C5—H5B	109.5	C8—C13—H13	119.6
H5A—C5—H5B	109.5	C10—C14—H14A	109.5
C4—C5—H5C	109.5	C10—C14—H14B	109.5
H5A—C5—H5C	109.5	H14A—C14—H14B	109.5
H5B—C5—H5C	109.5	C10—C14—H14C	109.5
C4—C6—H6A	109.5	H14A—C14—H14C	109.5
C4—C6—H6B	109.5	H14B—C14—H14C	109.5
H6A—C6—H6B	109.5	C11—C15—H15A	109.5
C4—C6—H6C	109.5	C11—C15—H15B	109.5
H6A—C6—H6C	109.5	H15A—C15—H15B	109.5
H6B—C6—H6C	109.5	C11—C15—H15C	109.5
C2—C7—C8	134.7 (3)	H15A—C15—H15C	109.5
C2—C7—H7	112.6	H15B—C15—H15C	109.5
			
C4—O1—C1—O3	164.6 (2)	C1—C2—C7—C8	−8.5 (6)
C4—O1—C1—C2	−19.9 (4)	C3—C2—C7—C8	−179.5 (3)
O3—C1—C2—C7	−5.1 (5)	C2—C7—C8—C13	−31.0 (6)
O1—C1—C2—C7	179.9 (3)	C2—C7—C8—C9	151.5 (3)
O3—C1—C2—C3	165.6 (3)	C13—C8—C9—C10	3.9 (4)
O1—C1—C2—C3	−9.4 (4)	C7—C8—C9—C10	−178.4 (3)
C4—O2—C3—O4	−160.0 (3)	C8—C9—C10—C11	−4.5 (4)
C4—O2—C3—C2	21.6 (4)	C8—C9—C10—C14	177.0 (3)
C7—C2—C3—O4	1.7 (4)	C9—C10—C11—C12	1.6 (4)
C1—C2—C3—O4	−169.8 (3)	C14—C10—C11—C12	−180.0 (3)
C7—C2—C3—O2	180.0 (3)	C9—C10—C11—C15	−177.7 (3)
C1—C2—C3—O2	8.5 (4)	C14—C10—C11—C15	0.8 (5)
C1—O1—C4—O2	47.5 (3)	C10—C11—C12—C13	1.8 (5)
C1—O1—C4—C6	161.9 (2)	C15—C11—C12—C13	−178.9 (3)
C1—O1—C4—C5	−73.7 (3)	C11—C12—C13—C8	−2.5 (5)
C3—O2—C4—O1	−48.1 (3)	C9—C8—C13—C12	−0.4 (5)
C3—O2—C4—C6	−162.8 (2)	C7—C8—C13—C12	−177.8 (3)
C3—O2—C4—C5	73.8 (3)		

### Hydrogen-bond geometry (Å, °)

**Table:**

*D*—H···*A*	*D*—H	H···*A*	*D*···*A*	*D*—H···*A*
C6—H6C···O4^i^	0.96	2.58	3.447 (4)	151

Symmetry codes: (i) *x*, *y*+1, *z*.
